# Effects of Textured Insoles on Balance in People with Parkinson’s Disease

**DOI:** 10.1371/journal.pone.0083309

**Published:** 2013-12-12

**Authors:** Feng Qiu, Michael H. Cole, Keith W. Davids, Ewald M. Hennig, Peter A. Silburn, Heather Netscher, Graham K. Kerr

**Affiliations:** 1 Department of Neurology, Nanjing Brain Hospital affiliated with Nanjing Medical University, Nanjing, Jiangsu Province, China; 2 Movement Neuroscience Program, Institute of Health and Biomedical Innovation, Queensland University of Technology, Brisbane, Queensland, Australia; 3 School of Exercise and Nutrition Sciences, Queensland University of Technology, Brisbane, Queensland, Australia; 4 School of Exercise Science, Australian Catholic University, Brisbane, Queensland, Australia; 5 Centre for Sports Engineering Research, Sheffield Hallam University, Sheffield, United Kingdom; 6 Biomechanics Laboratory, University of Duisburg-Essen, Essen, Germany; 7 Centre for Clinical Research, University of Queensland, Brisbane, Queensland, Australia; 8 International Children’s Orthotic Laboratory, Brisbane, Queensland, Australia; VU University Amsterdam, Netherlands

## Abstract

**Background:**

Degradation of the somatosensory system has been implicated in postural instability and increased falls risk for older people and Parkinson’s disease (PD) patients. Here we demonstrate that textured insoles provide a passive intervention that is an inexpensive and accessible means to enhance the somatosensory input from the plantar surface of the feet.

**Methods:**

20 healthy older adults (controls) and 20 participants with PD were recruited for the study. We evaluated effects of manipulating somatosensory information from the plantar surface of the feet using textured insoles. Participants performed standing tests, on two different surfaces (firm and foam), under three footwear conditions: 1) barefoot; 2) smooth insoles; and 3) textured insoles. Standing balance was evaluated using a force plate yielding data on the range of anterior-posterior and medial-lateral sway, as well as standard deviations for anterior-posterior and medial-lateral sway.

**Results:**

On the firm surface with eyes open both the smooth and textured insoles reduced medial-lateral sway in the PD group to a similar level as the controls. Only the textured insole decreased medial-lateral sway and medial-lateral sway standard deviation in the PD group on both surfaces, with and without visual input. Greatest benefits were observed in the PD group while wearing the textured insoles, and when standing on the foam surface with eyes closed.

**Conclusions:**

Data suggested that textured insoles may provide a low-cost means of improving postural stability in high falls-risk groups, such as people with PD.

## Introduction

Somatosensory feedback plays an important role in balance control and decreased somatosensory function, due to ageing and disease, has been closely associated with impaired mobility and falls in older people [1] and individuals with Parkinson’s disease (PD) [2]. People with PD may have reduced peripheral sensation arising from degeneration of cutaneous receptors and peripheral sensory nerves [3]. Furthermore, previous research has shown that PD fallers display reduced touch sensitivity [4] and greater levels of anterior-posterior [4,5] and medial-lateral [5] postural sway than PD non-fallers.

Many previous studies have attempted to alter the quality of the somatosensory information received from the feet to determine how it affects postural stability. Methods have included ischemic hypoxia of the foot, induced by a pressure cuff placed around the calcaneus [6], immersion of feet in iced water and standing on a foam surface [7-9]. All of these techniques have been shown to decrease or alter the somatosensory input from the foot and ankle and cause postural instability. 

Given the apparent relationship between reduced somatosensory function and poorer postural stability, it is unsurprising that numerous methods have been developed to enhance somatosensory feedback and improve postural stability. Artificially enhancing cutaneous feedback via mechanical vibration devices has been shown to reduce postural sway in healthy older adults [10,11]. Other research has shown that smooth strips of athletic tape placed across both ankle joints can provide additional somatosensory feedback to improve postural control regulation [12,13]. 

Our research [14] has also suggested that passive devices may provide an inexpensive and effective alternative to decrease postural sway in healthy older people. In a recent study by Hatton et al. [15] it was reported that mediolateral sway was decreased in older people when standing on textured surfaces. Similarly, Palluel et al. [16] reported reduced postural sway during quiet stance for older people wearing sandals with firm rubber nodules and Corbin et al. [17] observed reduced postural sway in younger participants while wearing insoles with a textured pattern. The data from these studies suggest that artificially-enhancing somatosensory information from the feet may be effective in improving standing balance in people with balance impairments. However, little work has been conducted to test the efficacy of enhanced somatosensory input on balance in PD patients [18,19]. For this reason we sought to examine standing postural control in samples of healthy older individuals and people with PD while wearing different insoles, under different visual conditions, on more and less stable surfaces. If it can be substantiated that balance in healthy older and PD individuals can be improved by adding texture to insoles, simple and inexpensive interventions can be developed for use in their daily activities to improve somatosensory function and benefit postural control to prevent falls. 

## Method

All experimental procedures on participants were approved by the Queensland University of Technology Human Research Ethics Committee. 

### Participants

Twenty people (13 males and 7 females; mean age 65±9 yrs) with Parkinson’s disease were recruited as participants in this study. They had a clinical diagnosis of idiopathic PD and were on a stable medication regime. The Unified Parkinson’s Disease Rating Scale (UPDRS) [20] and the Hoehn and Yahr Scale [21] were administered to quantify disease severity. An age- and gender-matched control group of 20 healthy older people (13 males and 7 females; mean age 69±5 yrs) was also recruited. Participants from both groups were required to be free of signs of dementia according to the Addenbrooke’s Cognitive Examination (Total score <82 out of 100) [22] and be free from serious co-morbidities or acute illnesses that would interfere with static standing or dynamic motion. The cohort number was determined to provide sufficient sample size based on previous studies of effects of textured insoles [14-17].

### Experimental Protocol

To examine postural stability under static conditions, postural sway was assessed for each participant using a force plate (OR6-6-2000, AMTI, USA). Centre of pressure data were recorded at 1000Hz for two, 30-second standing trials performed under each of the following conditions: 1) on a firm (more stable) surface with eyes open; 2) on a firm surface with eyes closed; 3) on a foam (less stable) surface with eyes open; and 4) on a foam surface with eyes closed. For assessment of postural sway on the foam surface, participants were asked to position themselves in the centre of a medium density foam block (74.5x62x15.7cm) that was positioned over the surface of the force platform (firm surface). This test battery was repeated three times in a random order to allow for the assessment of the three different footwear conditions: 1) barefoot; 2) wearing smooth insoles; and 3) wearing textured insoles. While standing on the force plate, participants were asked to look straight ahead at a cross positioned at eye level, with their feet 10cm apart and their hands at their sides. Measurements derived from centre of pressure displacements included the range of anterior-posterior (AP) and medial-lateral (ML) displacements and AP and ML standard deviation (SD).

**Figure 1:**
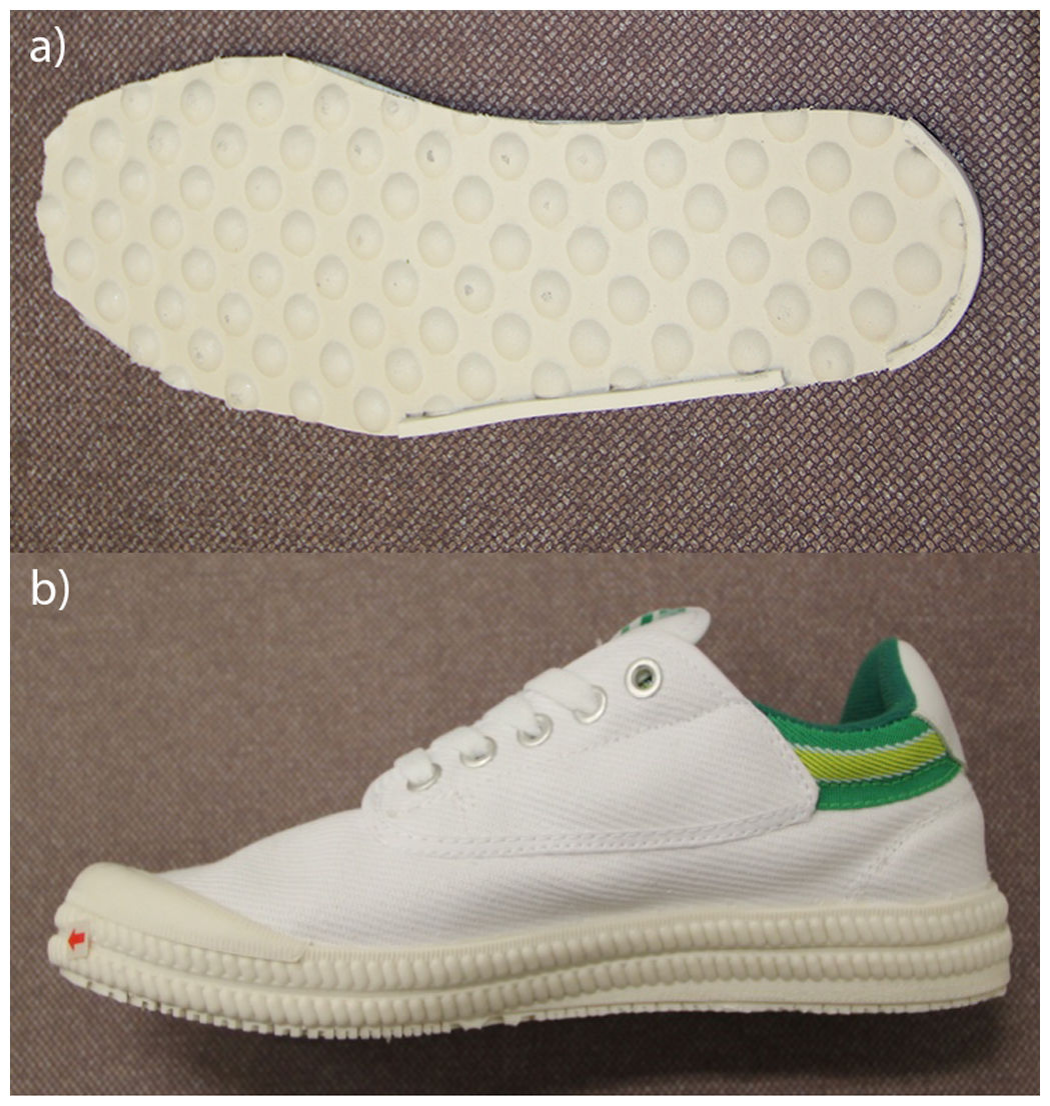
The characteristics of the textured insole (a) and the basic construct rubber-soled shankless shoe (b) used in this research.

The textured insoles used in this study were 1.5mm thick and constructed using soft insole material (270 density Ethylene Vinyl Acetate (EVA)) (International Children’s Orthotic Laboratory, Brisbane, Australia). The textured surface comprised granulations measuring 5.0mm in diameter and 3.1mm in height that were distributed evenly across the upper surface [14]. Similar to previous studies [23], the texture was accentuated by two raised compliant ridges measuring 3.1mm in height and 3.1mm in width which were located around the lateral perimeter of the insole and around the heel of the foot. This design was adopted to maximise the benefit afforded by insoles in a population which has significant balance and gait problems (Figure 1a). The smooth insoles were constructed with the same materials and had the same height and dimensions as the textured insoles, but without texture and raised ridges. These were used to ensure a standard insole surface and compliance within the shoes and were intended to be equivalent to a “shoe-alone” condition. For assessment purposes, the insoles were inserted into standardized footwear (Donated by Pacific Brands Australia Pty Ltd), comprising a basic construct rubber-soled shankless shoe with a soft canvas upper (Figure 1b). 

### Statistical Analysis

A mixed model Analysis of Variance (ANOVA) with 1 between-participant (PD; older) and 3 within-participant factors, including footwear (barefoot; smooth insole; textured insole), vision (eyes open; eyes closed) and surface (firm; foam) was used to compare the effects of different insoles while standing under different vision and stability conditions. Violations of the sphericity assumption for repeated measures variables were checked using Mauchley’s test of sphericity. Post-hoc comparisons were undertaken using the Fisher’s Least Significant Difference (LSD) test. Statistical significance was set at the 95% confidence level (P<0.05). Data were analysed using the Statistical Package for Social Sciences (SPSS V17.0, Chicago, IL, USA).

## Results

All PD and control participants who volunteered for the project completed all assessments. According to the clinical tests of disease severity, the PD participants were deemed to be predominantly early stage (Table 1).

**Table 1:** Average (and SD) age, height, mass, body mass index (BMI) and clinical scores for the Parkinson's disease and control groups.

	Parkinson’s disease	Controls	P-value
	(n=20)	(n=20)	
Age (years)	64.9 (8.9)	68.9 (4.8)	0.08
Height (m)	1.71 (0.75)	1.71 (0.78)	0.61
Mass (kg)	75.4 (12.7)	83.6 (15.3)	0.07
Body Mass Index (kg/m^2^)	25.8 (4.4)	28.4 (4.6)	0.07
UPDRS score	26.6 (10.8)		
Hoehn and Yahr	1.4 (0.9)		
Mean daily levodopa dosage (mg)	345.8 (394.7)		

UPDRS, Unified Parkinson’s Disease Rating Scale

### Anterior-Posterior Postural Sway and AP Postural Sway SD

#### Main Effects

Increased AP postural sway (F(1,78)=15.092, P<0.001) and AP postural sway SD (F(1,78)=20.470, P<0.001) was observed in the PD group compared to the control group. Significant main effects for surface and vision demonstrated that there was greater AP postural sway when standing on a foam surface compared to a firm surface (F(1,78)=332.750, P<0.001), and when standing with eyes closed compared to eyes open (F(1,78)=65.406, P<0.001). Similarly, significant main effects for surface and vision showed that AP postural sway SD was greater when standing on the foam surface (F(1,78)=272.913, P<0.001), and when standing with eyes closed (F(1,78)=23.498, P<0.001). However, there was no significant main effect of insole (AP postural sway: F(2,156)=0.246, P=0.782; AP postural sway SD: F(2,156)=0.903, P=0.408)

#### Interactions

There was a significant Surface * Vision interaction for AP postural sway (F(1,78)=26.477, P<0.001) and AP postural sway SD (F(1,78)=25.36, P<.001). Post-hoc analyses demonstrated that there was no significant change in AP postural sway or AP postural sway SD while standing on the firm surface with eyes open and closed. However, both sway measures were significantly increased on the foam surface without visual input. There were no other significant two- or three-way interactions for either AP postural sway or AP postural sway SD and no significant Group*Surface*Vision*Insole interaction (AP postural sway: F(2,156)= 2.928, P=0.056; AP postural sway SD: (F(2,156)=2.328, P=0.101).

### Medial-lateral Postural Sway

#### Main Effects

There was increased ML postural sway for the PD group compared to the control group (F(1,78)=12.668, P=0.001). The significant main effects for surface and vision demonstrated that there was greater ML postural sway when standing on the foam surface (F(1,78)=194.203, P<0.001), and when standing with eyes closed (F(1,78)=7.589, P=0.007). There was no significant main effect of insole for ML postural sway (F(2,156)=2.324, P=0.101).

#### Interactions

There were significant Surface*Vision (F(1,78)=7.639, P=0.007) and Surface*Group (F(1,78)=10.600, P=0.002) interactions. There was also a significant Group*Vision*Insole (F(2,156)=3.993, P=0.02) three-way interaction. Clear differences in postural sway as a function of group, insole, vision and standing surface were revealed by a significant Group*Surface*Vision*Insole interaction for ML sway (F(2,156)=3.232, P=0.042). 

#### Firm Surface Eyes Open

**Figure 2:**
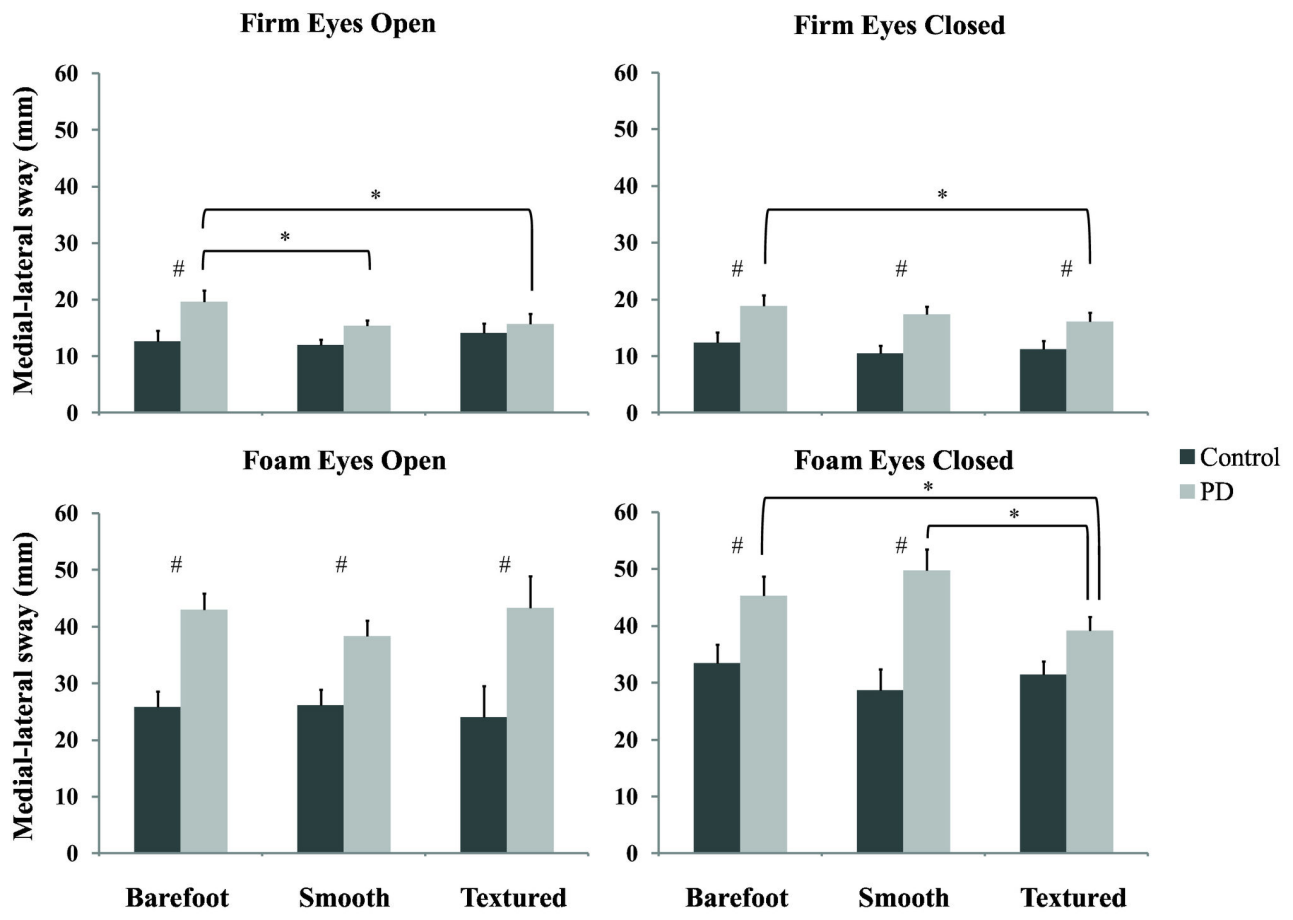
Mean (+1 SD) medial-lateral sway for the control (black) and PD (grey) participants under the four standing conditions. Note: # denotes a significant difference between the PD and control groups; * denotes a significant difference between the footwear conditions for the PD participants.

As shown in Figure 2, post-hoc analyses indicated that on the firm surface with eyes open, the PD group displayed greater ML postural sway than the control group while standing barefoot (P=0.012). Relative to the barefoot condition, both insoles decreased ML postural sway for the PD group (Fisher's LSD: Textured vs. Barefoot: P=0.011, Smooth vs. Barefoot: P=0.037, Textured vs. Smooth: P=0.830). This decrease was to a similar level as observed in the controls (PD vs Control: Smooth: P=0.127; Textured: P=0.515). 

#### Firm Surface Eyes Closed

Post-hoc analyses revealed that PD participants continually demonstrated significantly greater ML postural sway than controls under all three insole conditions on the firm surface with eyes closed (Barefoot: P=0.014; Smooth: P=0.004; Textured: P=0.026). Compared with the barefoot condition, the textured insole significantly decreased ML postural sway for the PD group (Fisher's LSD: Textured vs. Barefoot: P=0.011, Smooth vs. Barefoot: P=0.338, Textured vs. Smooth: P=0.304).

#### Foam Surface Eyes Open

Similarly, greater levels of ML postural sway were also evident for the PD participants on the foam surface with eyes open relative to observations of control participants (Barefoot: P<0.001; Smooth: P=0.003; Textured: P=0.015; Figure 2). Neither insole improved ML postural sway for control participants (F(2,77)=1.092, P=0.341). 

#### Foam Surface Eyes Closed

Under the least stable condition, standing on the foam surface with eyes closed, post-hoc analyses revealed that the PD group showed greater ML postural sway than the control group when barefoot (P=0.021) and with the smooth insole (P=0.001). In contrast, no difference was observed between the PD and control groups with the textured insole (P=0.052). Relative to the barefoot and smooth insole conditions, the PD participants demonstrated significantly reduced ML postural sway while wearing the textured insoles (Fisher's LSD: Textured vs. Barefoot: P=0.040; Textured vs. Smooth: P=0.001). There was no difference between the barefoot and smooth insole conditions for the PD participants (Fisher's LSD: P=0.141) (Figure 2). Furthermore, there were no significant differences between any of the insole conditions for the control participants (Fisher's LSD: Textured vs. Barefoot: P=0.485; Textured vs. Smooth: P=0.389; Smooth vs. Barefoot P=0.117).

### Medial-lateral Postural Sway Standard Deviation

#### Main Effects

Overall, ML postural sway SD was greater for the PD participants compared with the control participants (F(1,78)=13.165, P=0.001). A significant main effect of surface indicated that ML postural sway SD was greater when standing on the foam compared to the firm surfaces (F(1,78)=208.885, P<0.001). There was also a significant main effect of insoles (F(2,156)=5.825, P=0.004) and post-hoc comparisons identified that, compared with barefoot, ML postural sway SD was decreased while wearing the textured insoles (Fisher's LSD: P<0.001). ML postural sway SD was not significantly different between the smooth and textured insoles (Fisher's LSD: P=0.127) or between the smooth insoles and barefoot condition (Fisher's LSD: P=0.115). No significant main effect was evident between the eyes open and eyes closed conditions (F(1,78)=3.655, P=0.060). 

#### Interactions

There were significant Surface*Group (F(1,78)=10.559, P=0.002), Surface*Vision (F(1,78)=9.565, P=0.003), Surface*Insole (F(2,156)=3.23, P=0.042), and Group*Vision*Insole (F(2,156)=7.271, P=0.001) interactions. A significant Group*Surface*Vision*Insole interaction (F(2,156)=3.40, P=0.036) showed clear differences in the variability of postural sway under different conditions. No other significant interactions were obtained for ML postural sway SD.

#### Firm Surface, Eyes Open

**Figure 3:**
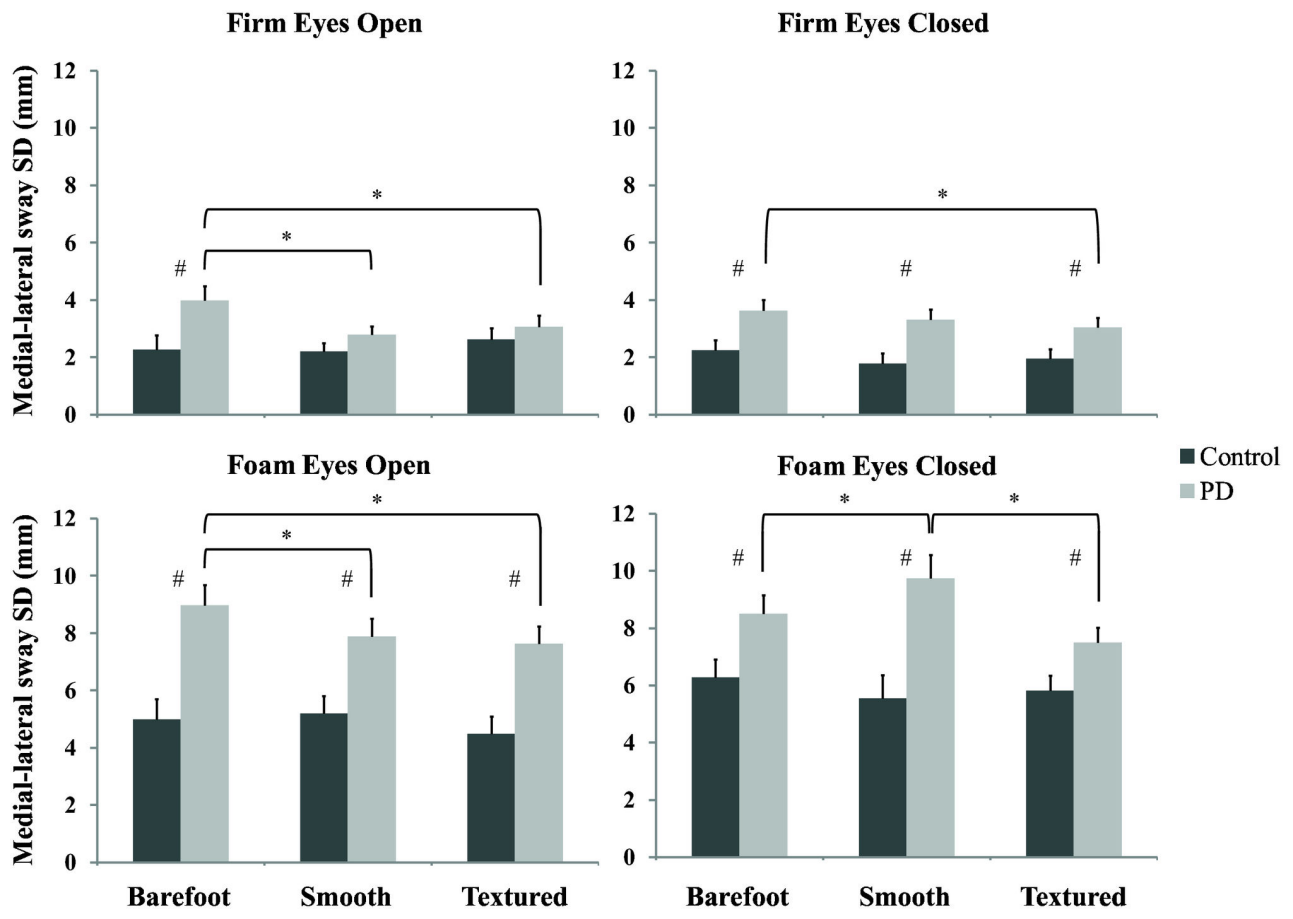
Mean (+1 SD) medial-lateral sway standard deviation for the control (black) and PD (grey) participants during the four standing conditions. Note: # denotes a significant difference between the PD and control groups; * denotes a significant difference between the footwear conditions for the PD participants.

As shown in Figure 3, on the firm surface with eyes open, the PD group displayed greater ML postural sway SD than the control group while barefoot (F(1,78)=5.758, P=0.019). However, both the smooth and textured insoles reduced ML postural sway SD in the PD group to a similar level as the controls (Smooth: F(1,78)=2.020, P=0.159; Textured: F(1,78)=0.587, P=0.446). 

#### Firm Surface, Eyes Closed

Post-hoc analyses showed that PD participants continually demonstrated significantly greater ML postural sway SD than controls under all three insole conditions while standing on a firm surface with eyes closed (Barefoot: P=0.008; Smooth: P=0.003; Textured: P=0.025). There was also a significant decrease in ML postural sway SD for the PD participants with the textured insoles compared to the barefoot condition (Barefoot vs Textured P=.004).

#### Foam Surface, Eyes Open

PD participants had greater ML postural sway SD than control participants for all insole conditions (Barefoot: P<0.001; Smooth: P=0.003; Textured: P<0.001). Both the smooth and the textured insoles reduced postural sway SD for the PD group under this condition (Barefoot vs Smooth P=0.029; Barefoot vs Textured P=0.003).

#### Foam Surface, Eyes Closed

In the least stable performance condition, standing on the foam surface with eyes closed, post-hoc analyses revealed that the PD group showed greater ML postural sway SD than the control group for all insole conditions (Barefoot: P=0.016; Smooth: P=0.001; Textured: P=0.022). However, for the PD participants, the results showed that wearing the textured insoles significantly reduced ML postural sway SD relative to the smooth insoles (Fisher's LSD: P=0.001), but not the barefoot condition (Fisher's LSD: P=0.058). When wearing the smooth insoles, ML postural sway SD was increased significantly compared to the barefoot (Fisher's LSD: P=0.049) and textured insoles (P=0.001) for the PD participants (Figure 3). 

## Discussion

The results showed that, while both insoles decreased ML postural sway and ML postural sway SD under specific conditions, the most significant benefits were observed in the PD group with the textured insole, which improved standing balance performance under more challenging conditions.

Consistent with previous research [24,25], the PD group displayed greater medial-lateral postural sway than the control group. Both insoles were effective in reducing ML postural sway in the PD group, but the efficacy of the insoles was clearly dependent on the type of support surface and the availability of vision. Both the smooth and textured insoles reduced ML sway for the PD group to a level equivalent to that of the control group when standing on a firm surface with unconstrained visual feedback. Conversely, the control group demonstrated no changes in postural sway under any of the insole conditions. Given that both insoles contributed to performance improvements for the PD group, it could be argued that the benefits observed in this study on the firm surface may have been due to the wearing of shoes rather than to the insoles themselves. Nonetheless, these findings are important because previous research has identified increased postural sway on a firm surface with eyes open to be a significant risk factor of falls for people with PD [4,5]. It is also important to recognise that the potential “shoe-alone” effect for data observed in the firm surface eyes open condition was not apparent in any of the other experimental conditions. One plausible explanation for this apparent selective influence is that participants may have been adapting to the relatively novel constraint of standing on the foam surface and re-prioritizing the somatosensory information to adapt to the different surface. This plausible explanation raises interesting possibilities that participants may selectively attend to (and prioritise) different task constraints in order to achieve an important goal of maintaining their stability and needs to be further tested in future research.

Under more challenging balance conditions, such as standing on a foam surface with eyes closed, the textured insoles were effective in reducing ML sway for the PD group to a level that was equivalent to the control group. The results for the firm surface revealed that the textured insoles provided no significant benefit to the control group with respect to ML sway. These findings suggested that, while standing on the foam surface without visual input, the raised surface of the textured insoles may have been effective in distributing increased pressure to the mechanoreceptors on the plantar surface of the foot. This additional stimulation could have resulted in enhanced somatosensory feedback to the central nervous system and contributed to significantly improved postural control [26,27].

In most experimental conditions, standing on the foam surface led to more ML postural sway than the firm surface, which was consistent with previous research [13,14,28]. The increase in sway was likely to be related to deficits in somatosensory information which would have resulted in participants having difficulties detecting the ground clearly; somatosensory feedback assumes increased importance when standing on an unstable foam surface [13,28]. The textured insoles may be effective in ameliorating some of these deficits in somatosensory function for the PD participants and demonstrated the most pronounced effect under conditions where there was greater reliance on somatosensory information (foam surface, eyes closed).

Overall, the textured insoles decreased postural sway and improved balance stability in the PD group most likely due to the enhancement of somatosensory information from the feet. Our results contribute to current understanding in the literature by complementing and disambiguating some of the extant data. For example, our findings are in agreement with data reported by Priplata et al. [11] who studied the use of insoles to propagate vibration to the plantar foot surface, demonstrating that postural sway can be decreased significantly during quiet standing through enhanced somatosensory feedback. However, such devices may be too cumbersome, complex and expensive for use in everyday life. Textured insoles may provide a viable alternative and act as an inexpensive way of improving postural stability. 

Our findings were consistent with previous work by Hatton et al. [15] and Qiu et al. [14] who showed that standing on a textured surface could decrease medial-lateral sway during standing in healthy older people. However, it is important to note that those research studies did not attempt to insert a textured insole into shoes, but rather studied the effects of a textured standing surface in healthy younger and older adults. 

Corbin et al. [17] and Palluel et al. [16,29] did report significant reductions in postural sway during standing balance while wearing textured insoles and sandals, respectively. However, Corbin et al. [17] only studied the effects of their textured insoles in a younger cohort, while the sandals evaluated by Palluel et al. [16,29] may not be suitable for all individuals and their use may be limited by health and safety requirements in some environments (e.g. workplace environments). Furthermore, Palleul et al. [16] maintained a consistent order of testing (“spike” followed by “without spike” – their Figure 2) which might have introduced a systematic order effect in their results. Previous research by Maki et al. [23] suggested that textured insoles were effective in improving postural stability by controlling lateral stability during walking in older people. In accordance with the findings presented by Maki et al. [23], the results of the current study showed a similar tendency for textured insoles to decease ML postural sway in people with PD in standing. A future study in our programme of work will examine the effects of this type of textured insoles on postural stability in both standing and walking, especially in people with PD and other populations with a higher risk of falling. The present investigation sought to maximise textured insole effects rather than compare different densities, compliance, nodule size or independent effect of ridges. Whilst this approach delimits the effects to our specific insole design, our current and future research is aimed at determining the relative effectiveness of different insole properties.

The time course of adaptation of participants to the textured insoles is also an important aspect of future research needed in this area. Palluel et al. [16,29] have reported changes over a 5-minute duration. However, effectiveness of insoles in supporting perceptual-motor function needs to be investigated over much longer timescales spanning several months. Whilst our participants reported that the insoles were comfortable to wear during the assessments, ensuring long-term adherence will require that comfort is assessed over extended periods of time. The data from our study are consonant with expectations from a dynamical systems perspective which has revealed multiple timescales of adaptation to task constraints, with clear predictions of large individual differences in the rates of adaptation by participants to new task constraints [30]. Overall there is a paucity of systematic research in this area to determine optimal texture characteristics for specific populations and task and environmental contexts.

To summarise, this study indicated that standing postural stability can be improved in people with PD, possibly by enhancing somatosensory information received from plantar mechanoreceptors of the feet. Textured insoles decreased both ML postural sway and ML SD under challenging conditions when standing on a foam surface with eyes closed. Such textured insoles may provide a low-cost means of improving postural stability in high-risk groups, such as people with PD, which may act as an important intervention to prevent falls.
